# The extracellular calcium-sensing receptor regulates human fetal lung development via CFTR

**DOI:** 10.1038/srep21975

**Published:** 2016-02-25

**Authors:** Sarah C. Brennan, William J. Wilkinson, Hsiu-Er Tseng, Brenda Finney, Bethan Monk, Holly Dibble, Samantha Quilliam, David Warburton, Luis J. Galietta, Paul J. Kemp, Daniela Riccardi

**Affiliations:** 1 School of Biosciences, Cardiff University, CF10 3AX, United Kingdom; 2 The Saban Research Institute, Children Hospital Los Angeles, 4650 Sunset Blvd # 83, Los Angeles, CA 90027, +1 323-362-5422, USA; 3 Laboratory of Molecular Genetics, Giannina Gaslini Institute, Genoa, Italy

## Abstract

Optimal fetal lung growth requires anion-driven fluid secretion into the lumen of the developing organ. The fetus is hypercalcemic compared to the mother and here we show that in the developing human lung this hypercalcaemia acts on the extracellular calcium-sensing receptor, CaSR, to promote fluid-driven lung expansion through activation of the cystic fibrosis transmembrane conductance regulator, CFTR. Several chloride channels including TMEM16, bestrophin, CFTR, CLCN2 and CLCA1, are also expressed in the developing human fetal lung at gestational stages when CaSR expression is maximal. Measurements of Cl^−^-driven fluid secretion in organ explant cultures show that pharmacological CaSR activation by calcimimetics stimulates lung fluid secretion through CFTR, an effect which in humans, but not mice, was also mimicked by fetal hypercalcemic conditions, demonstrating that the physiological relevance of such a mechanism appears to be species-specific. Calcimimetics promote CFTR opening by activating adenylate cyclase and we show that Ca^2+^-stimulated type I adenylate cyclase is expressed in the developing human lung. Together, these observations suggest that physiological fetal hypercalcemia, acting on the CaSR, promotes human fetal lung development via cAMP-dependent opening of CFTR. Disturbances in this process would be expected to permanently impact lung structure and might predispose to certain postnatal respiratory diseases.

During gestation, the developing lung goes from a fluid-filled organ in the fetus to a fully formed, highly mature structure equipped for optimal gaseous exchange from the moment of birth^1^,^2^,^3^. An impairment of the lung developmental programme *in utero* is at the basis of many respiratory diseases^3^,^4^. Lung development is characterised by five developmental different stages: embryonic, pseudoglandular, canalicular, saccular and alveolar. At the end of the embryonic stage, the lung is almost a compact structure with very small intraluminal volume and poor compliance. During the pseudoglandular stage (E11.5–16.5 in mice, weeks 5–17 in humans) there is a rapid expansion of the conducting airways when the developing lung undergoes stereotypic branching and budding^5^, a process which is driven by fluid secreted into the lung lumen to generate the distending pressure required for normal expansion^6^,^7^. Sustained reduction in lung expansion, such as that observed as a secondary consequence of failed closure of the diaphragm during fetal development leading to congenital diaphragmatic hernia^8^, contributes to fetal lung hypoplasia, which is highly associated with respiratory insufficiency at birth and respiratory distress syndrome during childhood^2^. On the other hand, increasing the intraluminal hydrostatic pressure by tracheal occlusion leads to an increase in lung DNA and protein content, and to lung hyperplasia^2^,^3^,^9^,^10^. Critical to the generation of the transpulmonary pressure gradient necessary for lung expansion is transepithelial Cl^−^ transport^7^,^11^,^12^,^13^,^14^ which, together with Na^+^ electrodiffusion, drives net water movement as a consequence of the osmotic pressure gradient^7^.

Fetal lung development occurs in a relatively hypercalcaemic environment. Free ionized extracellular calcium concentration ([Ca^2+^]_o_) in both the human and mice fetus is approximately 1.6–1.7 mM, compared to 1.1–1.3 mM in the adult^15^. Recently, we have demonstrated that this relative fetal hypercalcaemia is necessary for optimal prenatal lung fluid secretion^10^, an effect which is mediated by the extracellular calcium-sensing receptor (CaSR)^10^,^16^, a G protein-coupled receptor (GPCR) whose expression is developmentally regulated and confined to the prenatal mouse and human distal lungs^10^,^17^,^18^. A number of different chloride channels have been shown to be expressed in fluid-secreting fetal alveolar epithelial cells including members of the chloride channel (CLC) family^19^,^20^, the Ca^2+^-activated chloride channel TMEM16A^21^,^22^, bestrophin-1^23^ and the cystic fibrosis transmembrane conductance regulator (CFTR)^24^, but the exact mechanism by which activation of the CaSR leads to chloride-driven fluid secretion in the fetal lung lumen is currently unknown^1^.

In this study, we sought to examine how activation of the CaSR leads to increased fluid secretion and lung growth within the developing human lung. Because much of the work aimed at testing the contribution of each Cl^−^ channel to lung fluid secretion has been done using genetically modified mice, initially we compared the expression of a variety of chloride channels in the developing mouse and human fetal lungs using immunohistochemistry. Subsequently we investigated the effects of pharmacological (calcimimetics) and physiological (Ca^2+^_o_) CaSR activation on fluid secretion and identified the main Cl^−^ channels responsible for this process in chemically-defined, serum-free whole mouse and human lung explant cultures. Finally, the signalling machinery involved in CaSR-driven fluid secretion was examined using live imaging in an *in vitro* cell reporter system.

## Methods

### Ethical approval

Wild type C57Bl/6 mice were housed conventionally with a 12 h light:dark cycle and had free access to food and water. All animal procedures were approved by the UK Home Office and carried out in the UK in accordance with the Animal (Scientific Procedures) Act 1986.

Human fetal tissue was obtained from ethically-consented maternal donor medical termination at 7–11 week of pregnancy following the guidelines of the Polkinghorne and Department of Health reports with approval from South East Wales Research Ethics Committee^25^,^26^. Upon consent for termination, full written consent for the tissue was obtained from the maternal donor as part of the Medical Research Council (UK)-sponsored South Wales Initiative for Transplantation (SWIFT) program. Gestational age was first assessed by ultrasound and confirmed using morphometric parameters after therapeutic abortion.

### Immunohistochemistry and immunofluorescence

Human fetal (7–11 week post conception) or E12.5 mouse lungs were fixed in formalin overnight and subsequently embedded in paraffin. 5 μm thick sections were deparaffinised in xylene and rehydrated using a decreasing alcohol-water series (100%, 90%, 75% ethanol), followed by washing in distilled water. Fischer rat thyroid (FRT) cells stably transfected with CFTR were grown on sterilized glass coverslips of 13 mm diameter in 24 well plates. The culture medium was removed and the cells were fixed in 4% (w/v) PFA in PBS for 15 minutes.

The immunohistochemical protocol used was established and optimised for each species and antibody (see Table 1). Briefly, permeabilization with 1% SDS or heat-induced epitope retrieval was perform with either citrate buffer pH 6 or Tris/EDTA buffer, pH 9 to recover the antigenicity of the tissue. Endogenous peroxidase activity was quenched by incubating the tissue with 1% hydrogen peroxide in methanol. Non-specific antibody binding was prevented by incubating the slides with blocking solution for 1 hour at room temperature. Primary antibodies were diluted in blocking solution and applied to slides overnight at 4 °C. Negative controls were carried out through substitution of the primary antibody with either rabbit or goat serum. Protein immunoreactivity was detected using a horseradish-peroxidase conjugated secondary antibody (Dako UK, Cambridgeshire, U.K.; 1:200) applied for 1 hour at room temperature. Antigen-antibody binding was visualized with diaminobenzidine (Sigma-Aldrich), after which the slides were counterstained with haematoxylin. The slides were dehydrated using an increasing alcohol series (30 s in 75% ethanol, 4 min in 100% ethanol) and finally cleared in xylene before being mounted using DPX mounting medium (Depex-Polystyrene in xylene, Timstar laboratory Suppliers, Ltd, Marshfield Bank, U.K.). Slides were left to dry overnight and then photographed using a microscope attached to an Infinity 2-2C CCD camera (Lumenera, Ottawa, Canada) and/or scanned using a slide scanner (MIRAX SCAN, Carl Zeiss MicroImaging GmbH, Göttingen, Germany).

5 μm thick, paraffin embedded week 10 human fetal lungs were dewaxed and rehydrated as described above. Samples for immunofluorescence were washed in 50 mM NH_4_Cl in PBS to quench free aldehyde groups remaining from the fixation step. Permeabilization and/or heat-induced epitope retrieval was performed as described above. Non-specific antibody binding was prevented by incubating the slides with blocking solution for 1 hour at room temperature.

Primary antibodies were diluted in blocking solution and applied to slides overnight at 4 °C. After washing in PBS, primary antibody binding was visualized using Alexa Fluor 594 fluorescence-dye coupled secondary antibodies in blocking buffer. Nuclei were counterstained with Hoechst 34580. Coverslips with adhering cells were then mounted on standard glass slides using ProLong Gold® (Life Technologies), while human lung slide were mounted in DPX after dehydration. Negative controls were carried out through substitution of the primary antibody with either rabbit or goat serum. Slides and cells were left to dry overnight and then epifluorescence images of immunostained tissues and cells were analysed and photographed with an Olympus BX61 automated microscope equipped with a 100 W high pressure mercury lamp, using AnalySIS software (Olympus Microscopy, Essex, U.K.).

### Lung explant culture

Lungs explanted from 7–9 week human fetal tissue and E12.5 mice were cultured for 48–72 hours according to previous published protocols^10^,^27^,^28^,^29^. Human fetal lungs were separated into two halves with the trachea alternatively kept with the left or right lung in the same experimental series, a manoeuvre which did not affect lung growth. Timelapse images were captured at 0, 24, 48 and 72 hours with a dissecting microscope equipped with a digital camera (Leica Microsystems, Milton Keynes, U.K.).

For the fetal Ca^2+^_o_ conditions, [Ca^2+^]_o_ in the DMEM-F12 medium was increased from 1.05 mM [Ca^2+^]_o_ to 1.70 mM [Ca^2+^]_o_ using 1 M CaCl_2_ (Sigma-Aldrich, Gillingham, U.K.). Inhibitors of chloride-transporting mechanisms included the wide-spectrum anion exchange inhibitor 4,4′-Diisothiocyano-2,2′-stilbenedisulfonic acid (DIDS) (Sigma-Aldrich) and the CFTR specific inhibitor Inh-172 (Tocris) and the loop diuretic, bumetanide (Sigma-Aldrich). All chloride channel inhibitors were dissolved in DMSO, which has previously been shown not to affect lung explant growth^10^. Vehicle control experiments were performed by adding the equivalent amount of DMSO to the lung cultures.

### Measurements of lung fluid secretion

Transepithelial electrical potential difference (PD), indicative of anion-driven fluid secretion into the lumen of the developing lung, was recorded as previously described^10^ after 48 or 72 hours for lung explant cultures for mouse and human lung, respectively. Briefly, lung explants attached to filters were placed in a recording chamber mounted on an Olympus CK41 inverted microscope (Olympus, Southall, U.K.) and secured using a slice anchor (Warner Instruments, Hamden, CT, USA). The recording chamber was filled with a solution containing (in mM): 135 NaCl, 5 KCl, 1.2 MgCl_2_, 1 CaCl_2_, 5 HEPES, 10 glucose, pH 7.45. A 2–3 MΩ borosilicate glass electrode (World Precision Instruments, Stevenage, U.K.) was filled with a solution containing a 0.4% trypan blue/0.85% saline solution (Invitrogen). Electrodes were slowly pushed into a terminal lumen whilst maintaining positive pressure, with the presence of trypan blue in the lumen being indicative of successful access to the lumen. Once access to the lumen had been achieved, positive pressure was immediately removed and the PD was recorded after a 5 min equilibration period. E12.5 mouse lungs were cultured for 48 h in the presence of 1.05 mM or 1.70 mM Ca^2+^_o_ in the presence or absence of the wide-spectrum anion exchange inhibitor which blocks Ca^2+^-activated chloride channels including TMEM16A and bestrophin, 4,4′-Diisothiocyano-2,2′-stilbenedisulfonic acid (DIDS)^1^,^30^,^31^, of the loop diuretic bumetanide, or of the selective CFTR inhibitor (Inh-172). Unless stated otherwise, transepithelial PD measurements were expressed as percentage of 1.05 mM Ca^2+^_o_ control.

### Measurements of CFTR activation using a halide-sensitive reporter system

CaSR-dependent activation of CFTR was measured by single cell imaging of Fischer rat thyroid (FRT) cells stably transfected with the human CFTR channel and a halide sensitive YFP (YFP-H148). The FRT-CFTR-YFP cell line has previously been used for high-throughput screening for the identification of CFTR modulators^32^.

FRT-CFTR-YFP cells were maintained in Coon’s modification of F12 (Sigma-Aldrich) supplemented with sodium bicarbonate (3.2 mM), 2 mM glutamine, 100 U/mL penicillin, 100 μg/mL streptomycin and 10% fetal bovine serum (Invitrogen). Cells were plated on to 13 mm glass coverslips 48 h before being placed in the light path of an inverted microscope (Olympus IX71, Southend-on-Sea, U.K.). A Xenon arc fluorescence light source was used to excite the intracellular YFP at 470 nm and visualise emission at 520 nm.

Cells were equilibrated using standard Dulbecco’s PBS (137 mM NaCl, 2.7 mM KCl, 8.1 mM Na_2_HPO_4_, 1.5 mM KH_2_PO_4_, 1 mM CaCl_2_ and 0.5 mM MgCl_2_, pH 7.4) in presence of pharmacological agents including the adenylate cyclase activator forskolin (20 μM), the specific CaSR allosteric activator NPS-R568 (1 μM), the CFTR inhibitor Inh-172 (10 μM) and the adenylate cyclase inhibitor MDL-12330A (25 μM). During the experiments cells were pre-incubated with Dulbecco’s PBS in the presence and absence of various activators and inhibitors for at least 5 min before being perfused with Iodide-rich Dulbecco’s PBS (137 mM NaI, 2.7 mM KCl, 8.1 mM Na_2_HPO_4_, 1.5 mM KH_2_PO_4_, 1 mM CaCl_2_ and 0.5 mM MgCl_2_, pH 7.4) while YFP fluorescence was imaged. Iodide influx quenches the cells YFP fluorescence with a rate that is dependent on the halide permeability of the cell, and therefore on the activity of the CFTR channel. Quenching rates were calculated by fitting the YFP fluorescence decay with a one-phase exponential decay function using GraphPad Prism 6.01 (GraphPad Software, La Jolla, CA, USA).

### Data analysis and statistics

Data are expressed as mean ± SEM. Significance was determined using a Student’s t test or one-way ANOVA with Tukey’s post-test. Statistical analyses were run in GraphPad Prism 6.01 and observations were deemed to be significantly different for p value < 0.05.

## Results

### Expression of Cl^−^ channels in the developing mouse and human fetal lungs

Our previous studies have demonstrated that CaSR expression is developmentally regulated in the fetal mouse and human lung, with expression peaking at around E12.5–13.5 for the mouse and weeks 8–11 in humans, during the pseudoglandular stage of lung development^10^,^18^.

We have also shown previously that, in intact mouse pseudoglandular lungs, CaSR activation leads to Cl^−^-driven fluid secretion by unknown mechanisms^10^. However, the relevance of these findings to human lung development and the molecular mechanisms underpinning this process are unknown. To answer these queries, we investigated the expression of key Cl^−^ channels putatively involved in anion-driven fluid secretion in the developing mouse and human lung *in vivo* during gestational stages of maximal CaSR expression.

**Figure 1:**
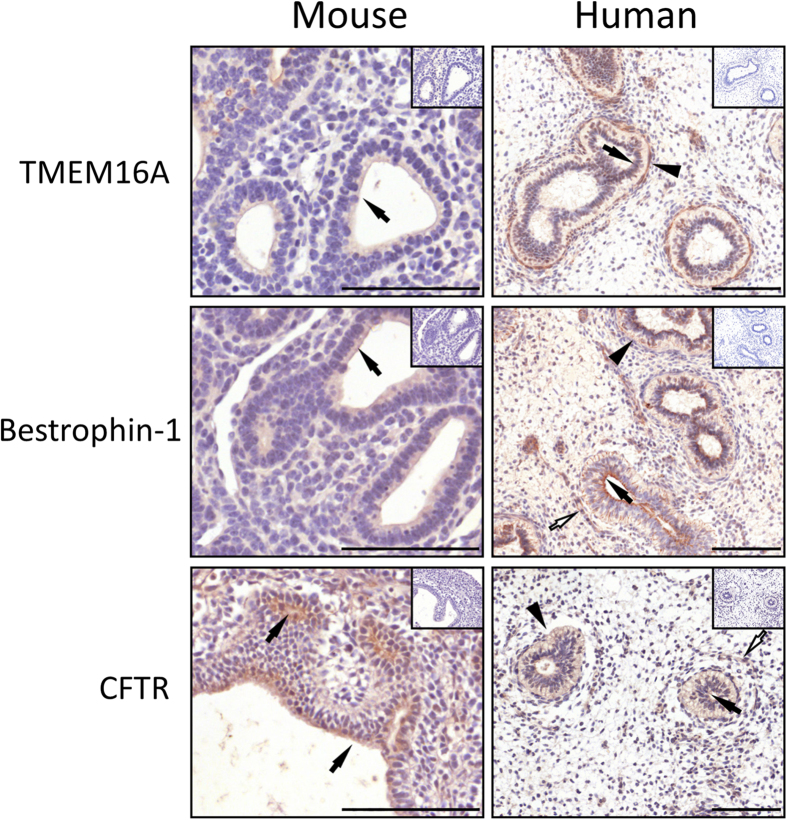
Expression of chloride channels in the developing mouse lungs and human fetal lungs. Paraffin-embedded, 5 μm-thick sections from E12.5 mouse (left panel) and week 9–11 human fetal lungs (right panel) were dewaxed and used for immunohistochemistry. Expression of the Ca^2+^-activated chloride channels TMEM16A, bestrophin-1 and CFTR were visualised using DAB (brown straining) in the lung epithelium. Sections were counterstained with Harris’ hematoxylin (blue staining). Negative controls were carried out in serial sections form the same lungs by substituting the primary antibody with an isotype control (inset). Block arrows show apical expression in the epithelium, arrowheads show basolateral expression and open arrows show expression in the mesenchyme. Scale bar = 100 μm.

Immunohistochemistry carried out on serial sections of E12.5 mouse (Fig. 1, left panels) and in human (Fig. 1, right panels) fetal lungs at 9–11 week gestation demonstrated that in both species the airway epithelium expresses the Ca^2+^-regulated Cl^−^ secretory channels, anoctamin-1/transmembrane member 16A (TMEM16A), bestrophin-1 and the cystic fibrosis transmembrane conductance regulator (CFTR). TMEM16A expression was detected apically in mouse fetal lungs (Fig. 1, left panel, block arrow) while in human fetal lungs TMEM16A appeared to be expressed predominantly at the basolateral domain of the pulmonary airways (Fig. 1, arrowhead) although modest staining was observed apically in small airways (Fig. 1, block arrow).

Bestrophin-1 expression was detected apically in the airway epithelia of mouse and human fetal lungs (Fig. 1, middle panels, block arrow). In the human lung, bestrophin-1 was also expressed in a sub-population of cells in the mesenchyme. Based on their arrangement and elongated appearance, these appear to be smooth muscles cells (Fig. 1, open arrow).

Strong apical CFTR expression was found in the developing mouse pulmonary airways (Fig. 1, bottom left panel, block arrow). Consistent with previous findings in human fetal lungs^24^,^33^, CFTR immunoreactivity was found apically and basolaterally in the pulmonary airways of human fetal lung at 10 week gestation (Fig. 1, bottom right panel, arrowhead). CFTR was also found in a discrete population of cells encircling the primitive lumen. These cells may be airway smooth muscle cells according to their arrangement and appearance, which is flattened and elongated (Fig. 1, left panel, open arrow). For all the antibodies tested negative controls, performed in serial sections, yielded no signals, as shown in the insets.

In the developing lung, basolateral Cl^−^ entry is mediated largely via the Na^+^/K^+^/2Cl^−^ co-transporter 1, NKCC1^31^,^32^, and indeed NKCC1 immunoreactivity was found at the basolateral side of the developing mouse and human lung (Fig. S1)^34^,^35^. In humans, NKCC1 immunoreactivity was detected in the columnar and cuboid epithelial cells lining the smaller airways. Furthermore, NKCC1 was present in the mesenchyme cells surrounding the human developing airways. Together, these results indicate that, in both human and mouse, bestrophin and/or CFTR are the most likely candidates for apical Cl^−^-driven fluid secretion. TMEM16A could also contribute to this process in the developing mouse, but not in the human lung.

### The apical Cl^−^ channels, TMEM16A and bestrophin-1, do not contribute to Ca^2+^_o_-stimulated lung fluid secretion in the mouse fetal lung

To elucidate which mechanisms are involved in CaSR-mediated fluid secretion, we measured transepithelial potential difference (PD) as a surrogate for anion-driven fluid secretion. Thus, E12.5 mouse lungs were cultured in the presence of blockers of those Cl^−^ channels found to be expressed at the apical membrane of lung lumen epithelial cells of the developing mouse lung, namely TMEM16A and bestrophin-1. Lungs cultured for 48 hours in the presence of medium containing 1.05 mM Ca^2+^_o_ in the absence or presence of DIDS (100 μM) appeared to be comparable in morphology and transepithelial PD (Fig. S2, 100 ± 44% in control conditions, 95 ± 27% in the presence of DIDS, n = 5–11, ns). Culturing E12.5 mouse lungs for 48 hours in the presence of medium containing 1.70 mM Ca^2+^_o_ led to an increase in transepithelial PD to 181 ± 29% (Fig. S2, n = 6, p < 0.01 vs. 1.05 mM Ca^2+^_o_ control). DIDS did not significantly alter the transepithelial PD when added to medium containing 1.70 mM Ca^2+^_o_ (Fig. S2, 225 ± 43%, n = 6, not significant (n.s.), p > 0.05). Figure S2 also shows that DIDS did not affect fluid secretion of mouse lungs cultured in the presence of ether 1.05 mM Ca^2+^_o_ or 1.70 mM Ca^2+^_o_. This result suggests that neither TMEM16A nor bestrophin-1 significantly contribute to Ca^2+^_o_ -stimulated fetal mouse lung fluid secretion.

Furthermore, we tested the contribution that CaSR makes to the regulation of basolateral Cl^−^ entry by measuring transpetithelial PD in lungs cultured in the presence of the loop diuretic bumetanide, an NKCC1 blocker. Lungs cultured in medium containing 1.05 mM Ca^2+^_o_ showed a decrease in luminal PD to increasing concentrations of bumetanide, with luminal PD reduced by ~50% compared to 1.05 mM Ca^2+^_o_ alone (Fig. S3 n = 16–23, p < 0.001). Similar to that which we have reported previously^10^, culturing E12.5 lung in medium containing fetal Ca^2+^_o_ concentrations (i.e., 1.70 mM) increased transepithelial PD compared to lungs cultured in the presence of medium containing 1.05 mM Ca^2+^_o_ control (Fig. S3, 124 ± 13% vs. 100 ± 15%, n = 23–29, p < 0.001). Culturing these lungs in the presence of bumetanide (30 μM) led to decreases in luminal PD to ~75% of the 1.70 mM Ca^2+^_o_ control (92 ± 10% vs. 124 ± 13%, n = 17–29, p < 0.001). These results suggest that basolateral Cl^−^ entry is not regulated by CaSR/Ca^2+^_o_.

### CFTR mediates CaSR-induced fluid secretion in the mouse and human fetal lung

**Figure 2:**
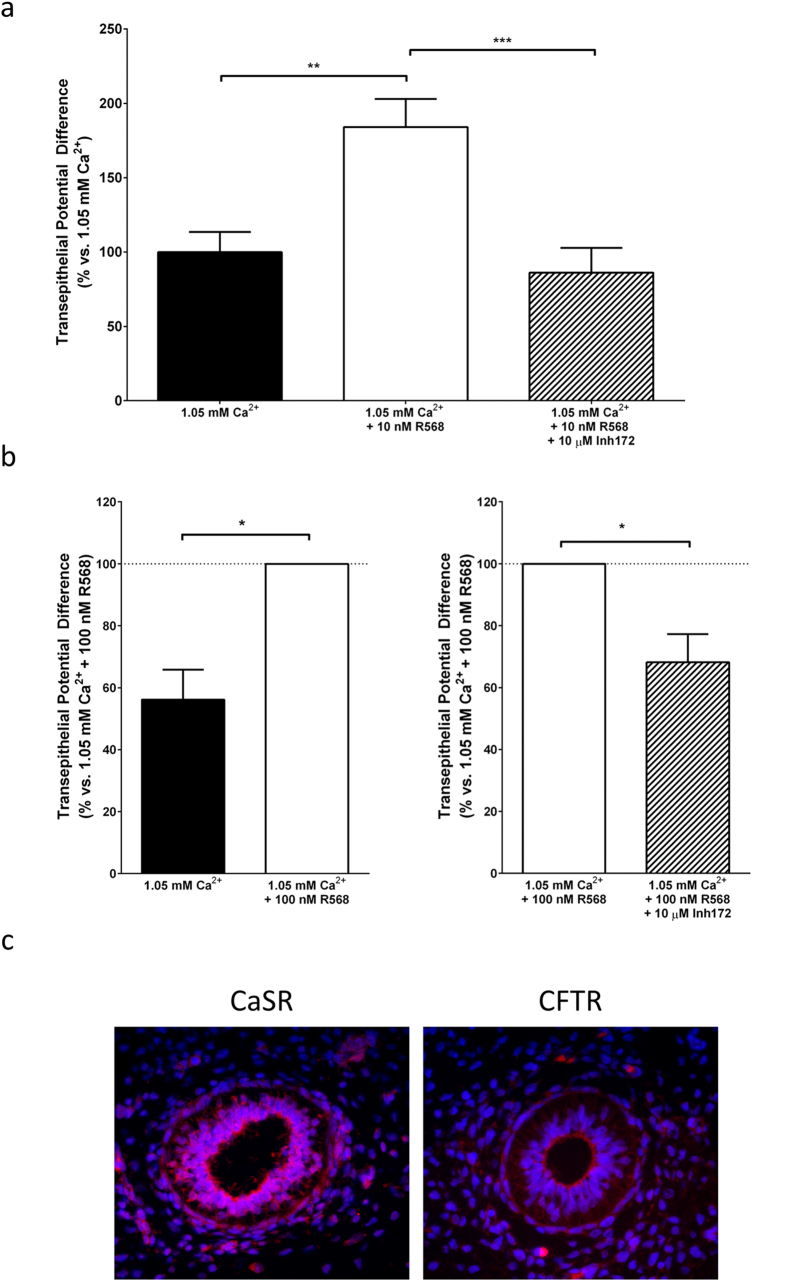
CaSR activation drives fluid secretion in fetal mouse and human lungs. (**a**) E12.5 lungs were cultured for 48 hours in the presence of medium containing either 1.05 mM, 1.05 mM Ca^2+^_o_ + the calcimimetic NPS-R568 or 1.05 mM Ca^2+^_o_ + NPS-R568 + the CFTR blocker, Inh-172, before measurement of transluminal potential differences were carried out. Pharmacological CaSR activation almost doubled the PD from 100 ± 13% to 184 ± 19%, effect which was completely abolished by Inh-172. Data were pooled from 3–4 separate isolations, n = 9, for all conditions and are presented as mean (as a percentage of 1.05 mM Ca^2+^_o_ control) ± SEM. **p < 0.01, one-way ANOVA with Tukey post-test. (**b**) Human lung rudiments were separated into two halves and then kept in culture for 72 h in medium containing either 1.05 mM Ca^2+^_o_ in the presence or absence of 100 nM NPS-R58 (n = 4), or 1.05 mM Ca^2+^_o_ + 100 nM NPS-R568 (n = 4) in the presence or absence of 10 μM Inh-172. Culturing human fetal lungs rudiments in the presence of 1.05 mM Ca^2+^_o_ + 100 nM NPS-R568 induced an increase in transluminal potential difference compared to its paired lung half cultured in 1.05 mM Ca^2+^_o_ alone. Furthermore, culturing fetal lung halves in the presence of medium containing 1.05 mM Ca^2+^_o_ + 100 nM NPS-R568 + 10 μM Inh-172 decreased transluminal potential difference in comparison to its paired 1.05 mM Ca^2+^_o_ + 100 nM NPS-R568 lung half. Data are presented as mean difference from 1.05 mM Ca^2+^_o_ + 100 nM R568 ± SEM. **p < 0.01, paired t-test. (**c**) CaSR and CFTR are co-localised in the human fetal lung epithelium. Week 10 gestation human fetal lungs were obtained from maternal donors. Sections from ethically consented week 10 human fetal lungs were incubated with anti-CFTR (1:200) or anti-CaSR antibodies (1:200) and immunoreactivities were detected using Alexa Fluor 594 goat anti-rabbit secondary antibodies (1:200). Staining indicates that both CFTR and CaSR are present in the columnar and cuboidal epithelium cells of the primitive airways of human fetal lung.

The expression studies reported previously suggest that CFTR might be involved in CaSR-stimulated apical Cl^−^-secretion. To test this hypothesis further, we performed electrophysiological recordings of mouse and human lung explant cultured in the presence or absence of the specific CaSR allosteric activator^36^, NPS-R568, with or without the CFTR inhibitor, Inh-172. Culturing E12.5 mouse lungs for 48 h in the presence of low (i.e., 1.05 mM) Ca^2+^_o_ medium containing NPS-R568 (10 nM) led to a significant increase in transluminal PD from 100 ± 13% (n = 9) to 184 ± 19% (Fig. 2a; n = 9, p < 0.01). Addition of the CFTR inhibitor Inh-172 (10 μM)^37^ significantly blocked this increase (Fig. 2a; 86 ± 17%, n = 9, p < 0.001). These results suggest that, in mouse lungs, CaSR promotes fluid secretion via activation of CFTR.

To examine whether, in the developing human lung, activation of the CaSR is also involved in this Ca^2+^_o_-driven fluid secretion via activation of CFTR, we performed two sets of paired experiments using right and left lobes of the same lungs and compared 1.05 mM Ca^2+^_o_ vs. 1.05 mM Ca^2+^_o_ + 100 nM R568 (to test for CaSR-mediated activation of fluid secretion); and another comparing 1.05 mM Ca^2+^_o_ + 100 nM R568 vs. 1.05 mM Ca^2+^_o_ + 100 nM R568 + 10 μM Inh-172 (to test for CFTR-dependent CaSR-mediated activation of fluid secretion). In both sets of experiments, transepithelial PD was normalised against the 1.05 mM Ca^2+^_o_ + 100 nM R568 condition. Human lung rudiments cultured in medium containing 1.05 mM Ca^2+^_o_ showed significantly reduced transepithelial PD compared to those cultured in 1.05 mM Ca^2+^_o_ + 100 nM R568 (Fig. 2b, 56 ± 10% vs. 100% (n = 4), p < 0.05) indicative that pharmacological CaSR activation almost doubled Cl^−^ fluid secretion also in the human fetal lung. This CaSR-mediated increases in transluminal PD was blunted in the presence of Inh-172 (Fig. 2b, 100% vs 68 ± 9%, p < 0.05), confirming that CaSR activation induces an increase in fluid secretion via activation of CFTR in human fetal lung. Immunofluorescence microscopy of pseudoglandular human fetal lung sections revealed that both CaSR and CFTR were co-expressed at the apical membrane of the bronchial epithelium, suggesting the possibility of a close functional interaction between these two proteins (Fig. 2c).

### Physiological fetal hypercalcemia activates the CaSR leading to an increase in CFTR-driven fluid secretion in the human, but not in the mouse fetal lung

**Figure 3:**
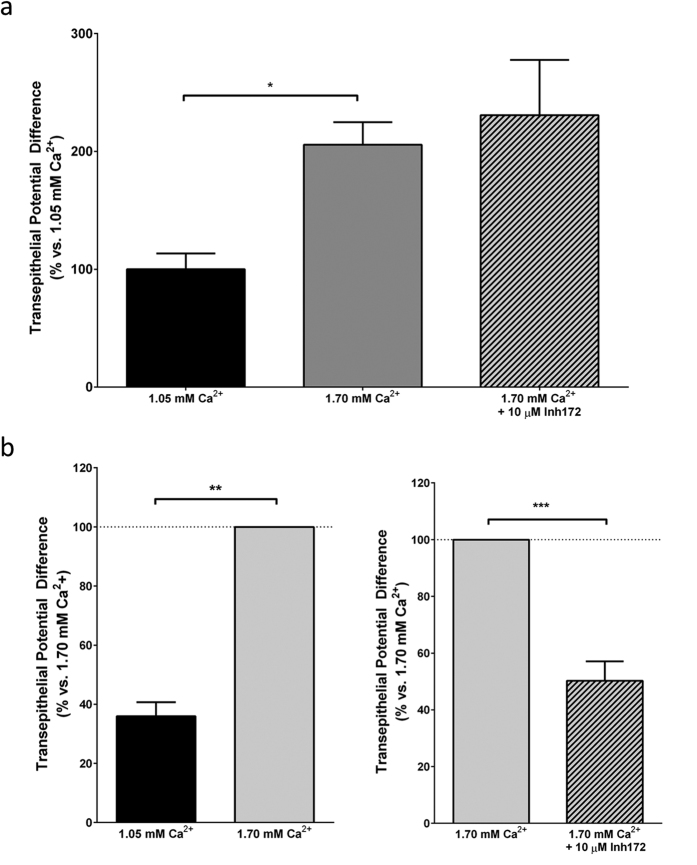
Fetal hypercalcaemia drives fluid secretion via activation of CFTR in the fetal human, but not mouse lung. (**a**) Inh-172 is a specific inhibitor for the CFTR channel. E12.5 lungs were cultured for 48 hours in the presence of medium containing 1.70 mM Ca^2+^_o_ with or without Inh-172, before measurements of transepithelial potential differences were carried out. Culturing E12.5 mouse lungs in the presence of Inh-172 did not significantly alter transepithelial potential differences in lungs cultured in medium containing 1.70 mM Ca^2+^_o_. (**b**) Human lung rudiments were separated into two halves and then kept in culture for 72 h in medium containing either 1.05 mM or 1.70 mM Ca^2+^_o_ in the presence or absence of the CFTR inhibitor, Inh-172. Culturing human lung rudiments in medium containing 1.70 mM Ca^2+^_o_ induced an increase in transluminal potential difference that was inhibited by Inh-172. Data are pooled from 4–6 different lungs for all conditions. Data are presented as mean difference from 1.70 mM Ca^2+^_o_ ± SEM. **p < 0.01, ***p < 0.001, paired t-test.

The fetus is relatively hypercalcemic compared to the mother, reaching Ca^2+^_o_ concentrations of ~1.7 mM, at which the CaSR is maximally active and that would lead to CaSR-mediated CFTR activation during developmental periods in development when the receptor is expressed. To test directly the hypothesis that fetal hypercalcemia, acting on the CaSR, provides a physiological stimulus for lung fluid secretion, we cultured E12.5 mouse lungs for 48 hours in medium containing either 1.05 mM or 1.70 mM Ca^2+^_o_ in presence or absence of Inh-172 (10 μM), before measurements of transepithelial PD were conducted. Lungs cultured for 48 hours in medium containing 1.70 mM Ca^2+^_o_ exhibited the expected increase in luminal PD to 221 ± 17% (Fig. 3a, n = 9, p < 0.05). However the addition of Inh-172 did not inhibit Ca^2+^_o_-mediated changes in transepithelial PD (Fig. 3b, 217 ± 38%, n = 10, n.s., p > 0.05). In paired human fetal lungs (i.e., one lung per condition from the same donor), lung rudiments cultured in medium containing 1.05 mM Ca^2+^_o_ showed a reduced transepithelial PD compared to 1.70 mM Ca^2+^_o_ (Fig. 3b, 40 ± 5% vs 100 (n = 4), p < 0.01). However, in stark contrast to what we observed in mouse, the addition of Inh-172 blocked this increase, as evidence by the drop in transepithelial PD from 100 to 50 ± % (Fig. 3b, n = 19, p < 0.001). Together, these results suggest that physiological fetal hypercalcemia stimulates fluid secretion via the CaSR in the developing human lung, a process which does not occur in the developing mouse lung, where hypercalcaemia drives anion-driven fluid secretion through channels other than CFTR.

### CaSR-mediated activation of CFTR requires adenylate cyclase

**Figure 4:**
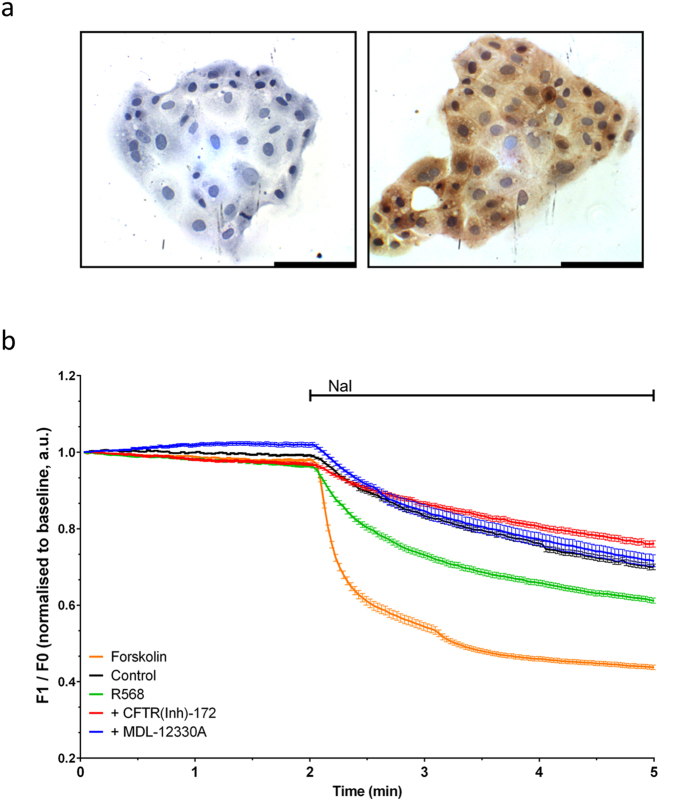
CaSR activation leads to opening of CFTR: involvement of a calcium-activated adenylate cyclase. (**a**) Fischer Rat Thyroid FRT cells, which endogenously express the CaSR, were engineered to express the CFTR channel and a halide-sensitive YFP as an indicator of channel opening. FRT cells were fixed with 4% paraformaldehyde before undergoing CaSR immunostaining. Primary antibody binding was detected using DAB (brown straining, right panel). Sections were counterstained with Harris’ hematoxylin (blue staining). Negative controls were carried out through the substitution of the primary antibody with an isotype control (left panel). Scale bar = 1000 μm. (**b**) FRT cells were pre-incubated for at least 2 min with NPS-R568 (1 μM) in the presence or absence of either the CFTR specific inhibitor, Inh-172 (10 μM) or the adenylate cyclase inhibitor, MDL-12330A (25 μM). 20 μM forskolin was used as a positive control, and pre-incubation with Dulbecco’s PBS was used as the time control. After 2 min the cell are perfused with iodide-rich Dulbecco’s PBS and the fluorescence of the halide-sensitive YFP is quenched at a rate dependent upon the halide permeability of the cell and therefore the activity of anion channels or transporters. The calcimimetic R568 quenched YFP fluorescence, demonstrating that CaSR activation in FRT cells leads to increased halide permeability of the cell. Inhibition of adenylate cyclase by MDL-12330A or CFTR by Inh-172 brought YFP quenching to levels similar to that of the time control – demonstrating that the increased halide permeability was due to activation of both adenylate cyclase and CFTR. (**b**) shows average traces of YFP quenching (±SD) over time from N = 6–13 separate experiments (n = 48–232 cells).

Having ascertained that, in the human fetal lung, CaSR and CFTR are functionally coupled, we investigated further the cellular mechanisms underpinning CaSR-mediated CFTR activation using an *in vitro* system previously used in high-throughput screening for identification of CFTR modulators^32^. Specifically, Fischer Rat Thyroid (FRT) cells, which here show to express endogenously the CaSR (Fig. 4a) were stably transfected with human CFTR and a halide-sensitive YFP probe (FRT-CFTR-hsYFP). Perfusion of iodide-rich PBS across FRT-CFTR-hsYFP cells led to a slight quenching of YFP fluorescence during the first three minutes due to the inherent halide permeability of the cell at a rate of k = 0.42 ± 0.02 (Fig. 4b, Table 2, N = 7, n = 197). When FRT cells were pre-incubated with forskolin to evoke an increase in intracellular cAMP levels^38^, a stimulus known to open CFTR^32^ (thereby acting as a positive control) the introduction of the iodide-rich DPBS led to a sharp decrease in YFP fluorescence at a rate of k = 1.76 ± 0.05 to 46 ± 2% (Fig. 4b, Table 2, N = 10, n = 150, rate: p < 0.001 vs. time control), indicating that the opening of CFTR led to an increase in the halide permeability of the FRT-CFTR-hsYFP cells.

To examine the effect of pharmacological CaSR activation on CFTR, FRT-CFTR-hsYFP cells were pre-incubated with the calcimimetic NPS R-568 (1 μM) before addition of the iodide-rich PBS. Pre-incubation with NPS R-568 led to a significant increase in the rate of YFP quenching compared to control, quenching YFP fluorescence to 61 ± 3% at a rate constant of k = 1.31 ± 0.03 (Fig. 4b, Table 2, rate: p < 0.001 vs. time control, N = 9, n = 168). Next, we determined whether this CaSR-mediated increase in halide permeability was due to opening of CFTR by pre-incubating the cells with NPS-R568 + Inh-172. This manoeuvre prevented CaSR-mediated activation of CFTR (Fig. 4b, Table 2, k = 0.42 ± 0.03, p < 0.001 vs. 1 μM NPS-R568, n.s. vs. time control, N = 8, n = 132). Similarly, cell pre-incubation with medium containing NPS-R568 + MDL-12330A, a pan-inhibitor of adenylate cyclase, also significantly reduced YFP quenching rates (Fig. 4b, Table 2, k = 0.56 ± 0.05, p < 0.001 vs 1 μM NPS-R568, N = 6, n = 93), demonstrating that the CaSR-mediated activation of CFTR requires activation of adenylate cyclase.

Classically CFTR is activated by cyclic AMP (cAMP)-dependent activation of protein kinase A (PKA). However, intracellular calcium ion (Ca^2+^_i)_-mobilising agonists are known to couple to Ca^2+^-activated chloride channels^39^. However, recently Ca^2+^_i_-mobilising agonists activate CFTR by a mechanism involving Ca^2+^_i_-dependent activation of adenylyl cyclase I (AC1) and cAMP/PKA signaling in human bronchial epithelial cells^39^. We have previously demonstrated in the developing mouse lung that CaSR activation leads to Ca^2+^_i_ mobilisation^10^ and therefore suggest that, in the human fetal lung, AC1 could also be involved in CaSR-mediated activation of CFTR. Indeed, AC1 is expressed within the columnar and cuboidal epithelial cells of the human fetal lung (Fig. S4). Therefore, CaSR activation, which yields an increase in Ca^2+^_i,_ would be expected to activate AC1, an effect which would result in opening of CFTR.

## Discussion

Fluid secretion into the lumen of the fetal lung is essential for optimal lung development as it provides the mechanical stress necessary for lung expansion, cell division and tissue remodelling^7^,^40^. Defective fluid secretion in the fetal lung leads to drastic changes in the developing organ structure^3^, with decreases in fluid secretion leading to hypoplastic lungs accompanied by alterations in lung morphology, whilst excessive fluid secretion produces hyperplastic and over-distended fetal lungs^41^,^42^, both of which are associated with long-lasting morbidity^3^. Previously, we have shown that physiological fetal hypercalcemia is an important environmental cue regulating many aspects of lung development^10^. Here we demonstrate that fetal hypercalcemia, acting through the CaSR, promotes human fetal lung growth and expansion via CFTR.

Apical Cl^−^ secretion results as a consequence of transepithelial Cl^−^ movements mediated by basolateral entry, raising its intracellular concentration above equilibrium facilitating its luminal exit through apical chloride channels^43^, in addition to isoosmotic transport of water^7^. Basolateral Cl^−^ entry occurs via NKCC1^7^,^44^ and inhibition of NKCC1 by the loop diuretic bumetanide inhibits lung liquid secretion in distal lung explants, an effect not seen in NKCC1 knockout mice^35^. Our study shows that NKCC1 is expressed on the basolateral membrane of columnar and cuboid epithelial cells in both developing mouse and human lungs and that, while bumetanide inhibited fetal lung fluid secretion by approximately 50%, this effect was insensitive to changes in Ca^2+^_o_ concentration, suggesting that this process is constitutively active and not regulated by the CaSR.

Our immunohistochemical observations indicate that apical Cl^−^ exit might be facilitated by TMEM16A, bestrophin-1 and CFTR in the developing mouse and human fetal lung. Additional channels and associated proteins involved in mediated calcium-activated chloride conductance - CLCN2 and CLCA1 - are also expressed in the developing human fetal lung at the basolateral membrane of epithelial cells and, as such, not likely to contribute directly to apical Cl^−^ exit (Fig. S5).

Previous studies have suggested that TMEM16A and bestrophin-1 may play important roles in generating the baseline fluid secretion^22^,^23^,^45^. However, the presence of DIDS in the growth medium had no effect on Ca^2+^_o_-mediated increases in fluid secretion suggesting that TMEM16A and bestrophin-1, as well as other DIDS sensitive Cl^−^ channels, do not appear to be involved in Ca^2+^_o_-activated fluid secretion.

These observations led us to conclude that CFTR is the primary apical Cl^−^ channel candidate functionally coupled to the CaSR. A role for CFTR in fetal lung development has been questioned because of conflicting findings between observations carried out in human and mouse lungs. On one hand, CFTR^−/−^ mice are born with apparently normal lungs^46^, suggesting either that CFTR plays no role in fetal lung fluid secretion or that there is functional redundancy. On the other hand, accumulating evidence in humans suggests that lungs of new-borns with cystic fibrosis (CF), an autosomal recessive disease caused by mutations leading to an inactive CFTR, have a number of functional and structural abnormalities^47^,^48^. These observations are consistent with the species differences seen in the current study. Of note, in humans CaSR and CFTR expression are developmentally regulated, both showing strongest expression within the pulmonary epithelium during the pseudoglandular stage of development^18^,^49^,^50^, the period critical for the formation of the conductive airways, corroborating the idea that the stimulatory role of the CaSR on fluid secretion could, indeed, be mediated by CFTR.

CFTR is insensitive to DIDS^51^. Therefore, we examined the effect of a specific CFTR inhibitor on CaSR-mediated fluid secretion in both human and mouse fetal lungs. The presence of Inh-172 in the growth medium suppressed increases in human and mouse fetal lung fluid secretion due to pharmacological CaSR activation. In human fetal lungs Inh-172 also suppressed Ca^2+^_o_-mediated increases in fluid secretion, suggestive that CaSR regulation of CFTR is the primary mechanism driving Ca^2+^_o_-mediated increase in fetal lung fluid secretion in humans. Importantly, inhibition of CFTR had no effect on Ca^2+^_o_-mediated increases in fluid secretion in the mouse fetal lung, suggestive that an alternative mechanism could account for Ca^2+^_o_-sensitive fluid secretion in mice, which could account for the species-dependent gating behaviour in murine and human CFTR^52^,^53^.

Conventionally CFTR is activated by cAMP and PKA whereas Ca^2+^-activated chloride channels are activated by Ca^2+^_i_-mobilising agonists like UTP. Furthermore, classical CaSR signalling is preferentially coupled to Gα_i_ leading to a decrease in intracellular cAMP^54^, which seems at odds with our finding that activation of CaSR leads to activation of CFTR^55^. Using co-immunoprecipitation pull-downs we found no evidence that the CaSR and CFTR have a biochemical interaction in either E12.5 mouse or 8 week human fetal lungs (*data not shown)*. However, pharmacological approaches demonstrated that the calcimimetic NPS R-568 induced opening of CFTR that was dependent on adenylate cyclase. Of the nine G-protein–responsive transmembrane adenylyl cyclase (AC1-9) isoforms identified, AC1 can be stimulated by Ca^2+^_i_/calmodulin in contrast to many other ACs, which are either Ca^2+^-insensitive or Ca^2+^-inhibited. In addition, Namkung *et al*. have showed that, in human bronchial cells, the Ca^2+^_i_ mobilising agonist, UTP, can stimulate CFTR opening through AC1^39^. In conjunction with our previous findings in fetal mouse epithelial buds^10^ that activation of the CaSR leads to Ca^2+^_i_ mobilisation, we propose that, in the developing human lung epithelium, CaSR activation by NPS R-568 and/or fetal hypercalcemia leads to an increase in Ca^2+^_i_, attendant activation of AC1 and opening of CFTR. A potentially similar mechanism has been described in duodenal epithelial cells, where CaSR agonists induce bicarbonate secretion via CFTR, through activation of PLC and increase in Ca^2+^_i _56.

Newborns with CF were long considered to be born with normal lungs^57^, with progressive structural abnormalities developing later^48^, suggestive that CFTR does not play a crucial role in lung developmental process. However, more recent studies have demonstrated that transient *in utero* disruption of CFTR leads to progressive changes in lung function and structure that appear to predispose to adult lung disease^58^,^59^, possibly due to interference in stretch-induced differentiation through inflammatory expression changes in smooth muscles proteins and/or the effects of a constitutive inflammatory process^58^,^60^,^61^. On the basis of our current findings, we suggest that mutations, which affect expression and/or function of CFTR, would lead to a reduction of increases in fluid secretion induced by fetal hypercalcemia via CaSR activation. In addition, respiratory problems, such as chronic and interstitial lung disease and reduction in gas exchange, observed in some patients with inactivating or activating mutations of the CaSR^62^,^63^,^64^,^65^,^66^ could also be caused by altered fluid secretion in the developing lung, which permanently compromises lung morphology and structure.

**Figure 5:**
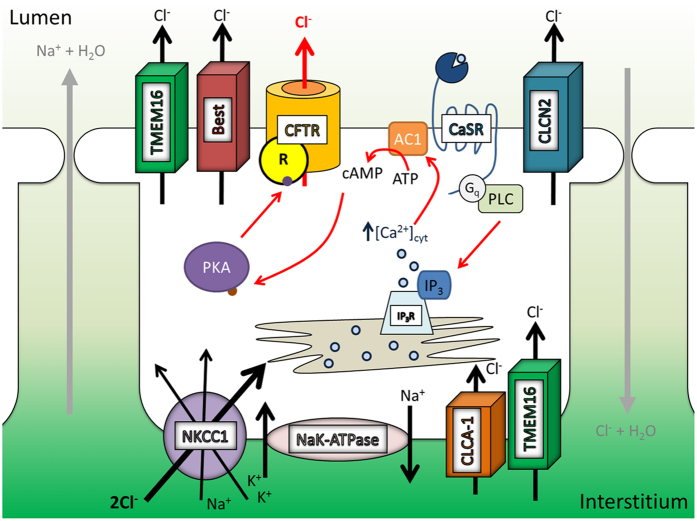
Proposed model for CaSR-mediated increase in fetal lung fluid secretion. In both the mouse and human activation of the CaSR via the calcimimetic NPS R-568 leads to an increase in cytosolic Ca^2+^ through activation of the PI-PLC pathway and release of Ca^2+^ from intracellular stores. This rise in cytosolic calcium leads to an increase in intracellular cAMP level via a Ca^2+^-stimulated adenylate cyclase (AC1), and in turn activation of the cAMP-dependent enzyme protein kinase A (PKA). Phosphorylation of the CFTR’s regulatory ‘R’-domain by PKA allows opening of the channel and conductance of Cl^−^ ions through the channel. A similar pathway also appears to be in place in response to fetal hypercalcemia in the human fetal lung, however this is not the case in the mouse where as yet unknown pathway, not involving an apical CFTR channel, appears to induce this increase in fluid secretion.

In conclusion, we have demonstrated that during the pseudoglandular stage of lung development fetal hypercalcemia of a magnitude similar to that seen physiologically in the prenatal period activates the CaSR leads to an increase in Ca^2+^_i_, which in turn activates the Ca^2+^-stimulated AC1, inducing opening of CFTR within the apical membrane of the developing pulmonary epithelium (Fig. 5). This does not occur in mice, which goes some way to explaining why human CF patients demonstrate persistent lung growth and developmental abnormalities while CFTR null mice do not. Finally, lung hypoplasia can result as a consequence of a wide variety of aetiologies. As catch up growth does not occur and damage to the lung developmental programme is permanent, stunted lung development is associated with long-lasting, deleterious effects and may predispose to many respiratory diseases. While fetal endoscopic tracheal occlusion has been used to reverse lung growth deficits^2^, it is only recommended in the most severe cases^67^, and shows partial success. Owing to their ability to promote CaSR-driven fluid secretion in the developing human lung, locally delivered calcimimetics could be used to increase the transmural pressure gradient and therefore offer the potential to rescue impaired growth in hypoplastic lungs *in utero*.

## Additional Information

**How to cite this article**: Brennan, S. C. *et al*. The extracellular calcium-sensing receptor regulates human fetal lung development via CFTR. *Sci. Rep.* **6**, 21975; doi: 10.1038/srep21975 (2016).

## Supplementary Material

Supplementary Information

## Figures and Tables

**Table 1:** Summary of primary antibody concentration, antigen retrieval solution and blocking solution for each protein.

	Primary Antibody	Antibody Concentration	Antigen Retrieval	Blocking Solution/Antibody dilution	Secondary Antibody	Company
CFTR	Polyclonal rabbit CFTR (ab59394)	Mouse (1:200) Human (1:200)	Citrate Buffer	3% NGS + 1% BSA	Goat anti-rabbit IgG-HRP (P0448)	Abcam
TMEM16A	Polyclonal rabbit TMEM16A (ab53212)	Mouse (1:200) Human (1:200)	Tris/EDTA	5% Seablock + 1% BSA		
Bestrophin-1	Polyclonal rabbit human Bestrophin (ab14927)	Mouse (1:200) Human (1:200)	Tris/EDTA	5% Seablock + 1% BSA		
CaSR	Monoclonal mouse CaSR (ab19347)	Human (1:200) FRT cells (1:100)	Citrate Buffer	5% Seablock in PBS	Rabbit anti-mouse IgG-HRP (P0260) IgG - Alex594 (A-11032)	
Adenylate Cyclase 1	Polyclonal rabbit AC1 (ac38331)	Human fetal lung (1:200)	Citrate buffer	5% Seablock in PBS	Goat anti-rabbit IgG-HRP	
NKCC	Polyclonal goat NKCC1 (sc21545)	Mouse (1:200) Human (1:50)	Tris/EDTA	5% Seablock in PBS	Donkey anti-goat IgG-HRP (sc2020)	Santa-Cruz Biotechnology
CLCA1	Polyclonal rabbit CLCA1 (sc67157)	Human (1:300)	1% SDS	5% Seablock in PBS	Goat anti-rabbit IgG-HRP	
CLCN2	Polyclonal rabbit CLCN2 antibody (ab49883)	Human (1:200)	Tris/EDTA	5% Seablock + 1% BSA		

**Table 2:** Rate of YFP Quenching and Quenching (%) for FRT-CFTR-hsYFP cells.

	Rate of Quenching (*k*)	Significance vs Control	Significance vs NPS-R568	Quench after 5 min (%)	Significance vs Control	Significance vs NPS-R568	
*Control*	0.42 ± 0.02			78 ± 3			N = 7, n =197
*1* *μM NPS-R568*	1.31 ± 0.03	p < 0.001		61 ± 3	p < 0.001		N = 9, n = 168
*1* *μM NPS-R568* + *10* *μM Inh-172*	0.42 ± 0.03	NS	p < 0.001	83 ± 1	NS	p < 0.01	N = 8, n = 132
*1* *μM NPS-R568* + *25* *μM MDL-12330A*	0.56 ± 0.05	p < 0.001	p < 0.001	78 ± 3	NS	p < 0.01	N = 6, n = 93
*20* *μM Forskolin*	1.76 ± 0.05	p < 0.001	p < 0.001	46 ± 2	p < 0.001	p < 0.001	N = 10, n = 150
