# Supplementary material for: The extracellular calcium-sensing receptor regulates human fetal lung development via CFTR
Source: Sci Rep. 2016 Feb 25;6:21975. doi: 10.1038/srep21975 (PMC4766410; doi:10.1038/srep21975)

Manuscript Title - The extracellular calcium-sensing receptor regulates human fetal lung development via CFTR

Authors: Sarah C Brennan, William J Wilkinson, Hsiu-Er Tseng, Brenda Finney, Bethan Monk, Holly Dibble, Samantha Quilliam, David Warburton, Luis J Galietta, Paul J Kemp and Daniela Riccardi

Supplementary Figures

Figure S1: Expression of NKCC1 in the developing mouse and human lung

Expression of NKCC1 was visualised using DAB (brown straining) in E12.5 mouse lungs (left panel) and week 9 -11 human fetal lung (right panel) epithelium. Sections were counterstained with Harris' hematoxylin (blue staining). Negative controls were carried out in serial sections by substituting the primary antibody with an isotype control (inset). Arrowheads show basolateral expression and open arrows show expression in the mesenchyme. Scale bar = 100 μm.

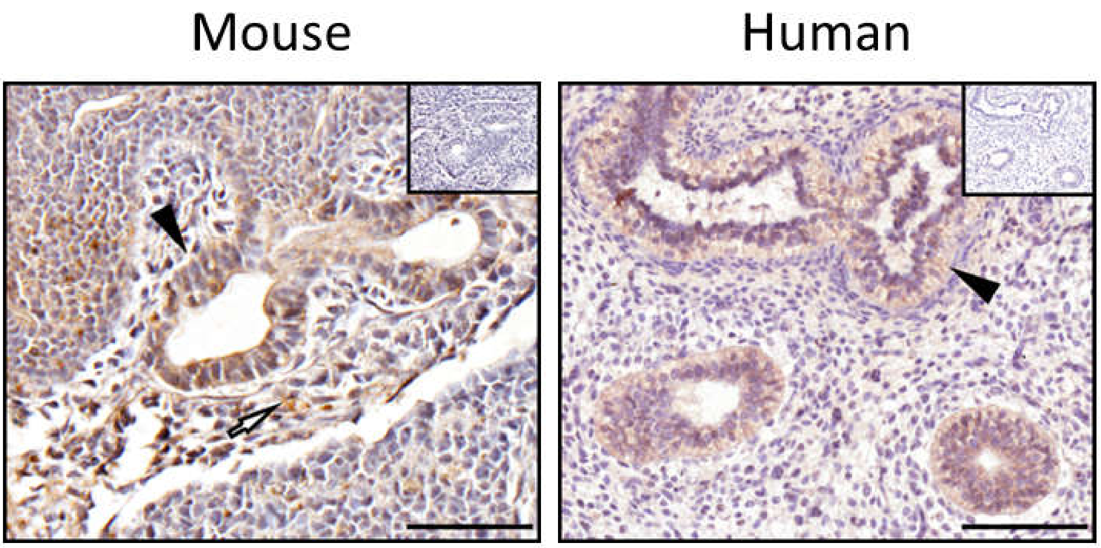

Figure S2: TMEM16A and bestrophin-1 do not contribute to on Ca^{2+}_o-stimulated fluid secretion

4,4'-Diisothiocyano-2,2'-stilbenedisulfonic acid (DIDS) is a wide-spectrum anion exchange inhibitor which blocks Ca^{2+}_o-activated chloride channels including TMEM16A and bestrophin-1. E12.5 lungs were cultured for 48 hours in medium containing either 1.05 mM or 1.70 mM Ca^{2+}_o in presence or absence of 100 µM DIDS, before transepithelial potential differences were measured using electrophysiological techniques. DIDS (100 µM) did not significantly affect fluid secretion in lungs cultured in medium containing 1.70 mM Ca^{2+}_o. Data were pooled from 3 separate isolations, n = 5 – 11, for all conditions and are presented as mean (as a percentage of 1.05 mM Ca^{2+}_o control) ± SEM. ns, $p > 0.05$, ** $p < 0.01$, one-way ANOVA with Tukey post test. *, 1.05 mM Ca^{2+}_o *vs.* 1.70 mM Ca^{2+}_o (post-test). Right panels, shows representative images of lungs at *t = 0* and *t = 48* h. Scale bar = 100 µm.

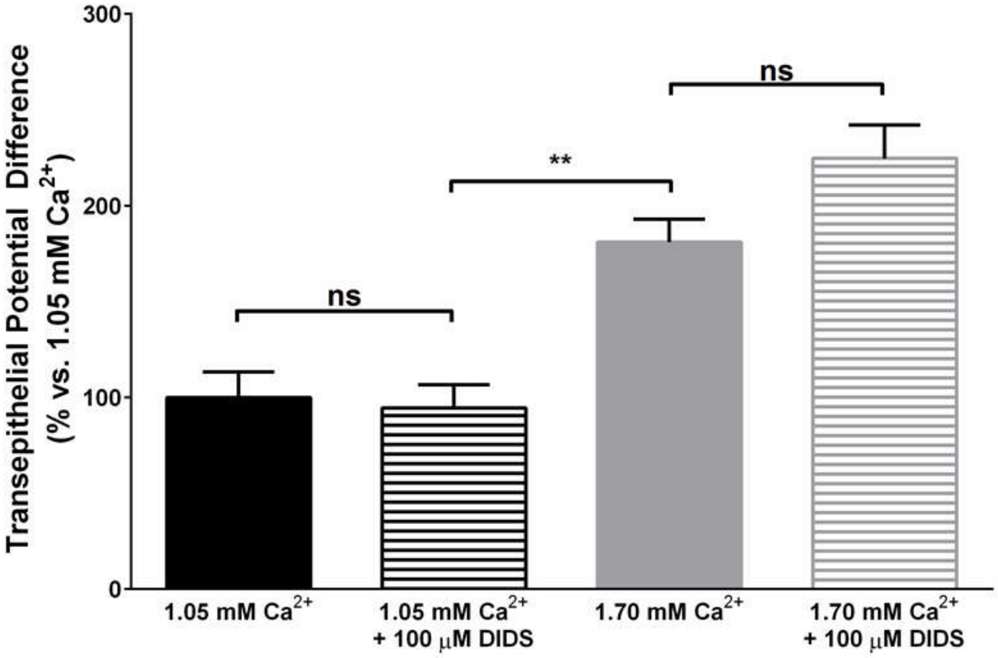

Figure S3. NKCC1-mediated fluid secretion is not modulated by Ca^{2+}_o

E12.5 lungs were culture for 48 h in the presence of medium containing either 1.05 mM or 1.70 mM Ca^{2+}_o in the absence or presence of the NKCC1 inhibitor, bumetanide (30 μM). Transepithelial potential differences were measured and then normalised to 1.05 mM Ca^{2+}_o control. Bumetanide decreased transepithelial potential difference to ~ 50 - 70% of the control in lungs cultured in medium containing either 1.05 mM or 1.70 mM $Ca^{2+}_{o.}$ Data were pooled from 5 – 6 separate isolations, n = 6 – 29, for all conditions, and are presented as mean (as a percentage of 1.05 mM Ca^{2+}_o control) ± SEM. ****/++++/#### $p < 0.001$, two-way ANOVA with Tukey post test, * 1.05 mM Ca^{2+}_o *vs*. 1.70mM Ca^{2+}_o (post-test), + 1.05 mM Ca^{2+}_o vs. 1.05 mM Ca^{2+}_o + bumetanide (post-test), # 1.70 mM Ca^{2+}_o vs. 1.70 mM Ca^{2+}_o + bumetanide (post-test).

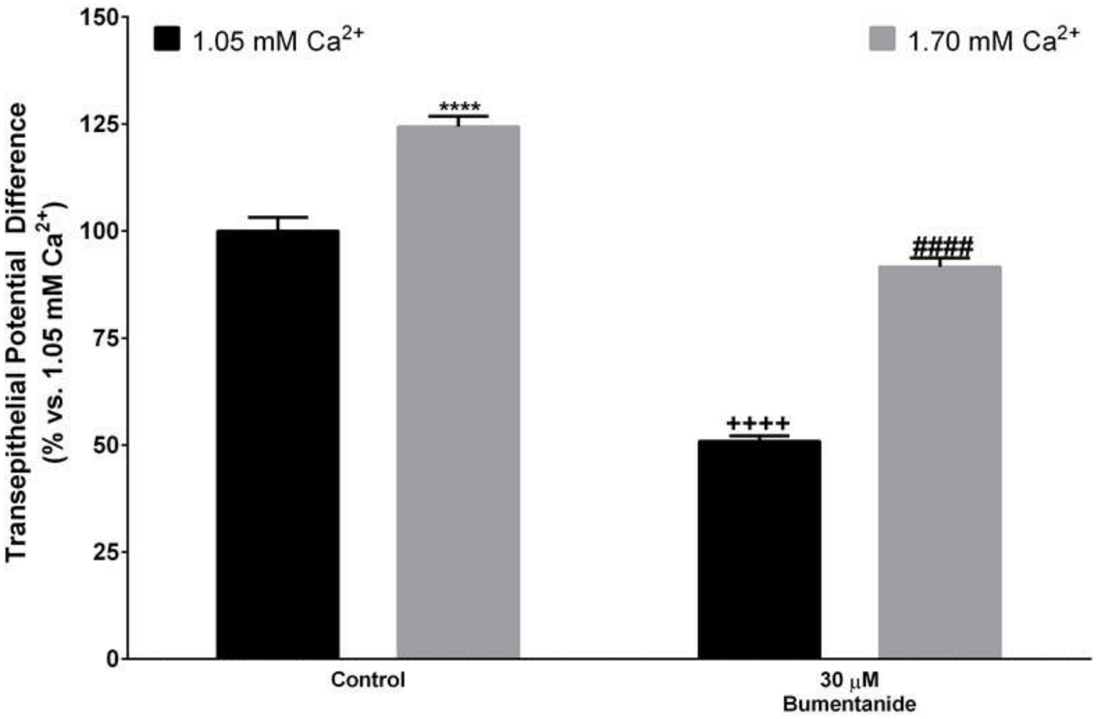

Figure S4. The Ca^{2+}-activated type I adenylate cyclase (AC1) is expressed in the human fetal lung

Paraffin-embedded, 5 µm-thick sections from week 10 human fetal lungs were dewaxed and used for immunohistochemistry. Expression of the Ca^{2+}-activated adenylate cyclase isoform I was visualised using DAB (brown straining; right panel) and is visible both apically and basolaterally in the developing human lung epithelium. Sections were counterstained with Harris' hematoxylin (blue staining). Negative controls (left panel) were carried out on serial sections by substituting the primary antibody with an isotype control. Scale bar = 1000 µm.

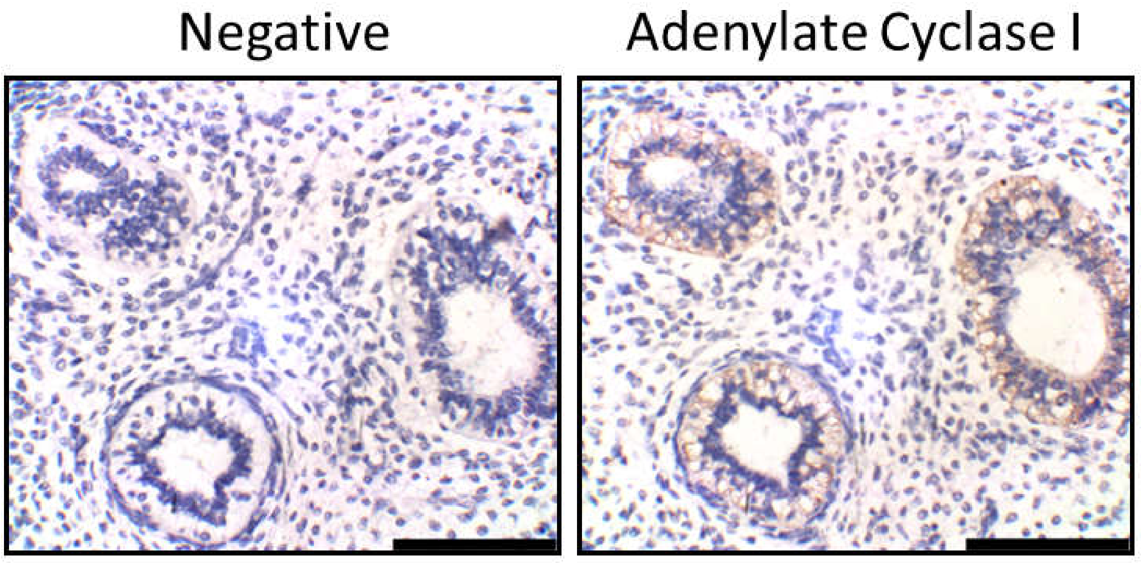

Figure S5. CLCN2 and CLCA1 are expressed at the basolateral side of the developing epithelium of the human fetal lung

Paraffin-embedded, 5 µm-thick sections from week 10 human fetal lungs were dewaxed and used for immunohistochemistry. Expression of the CLCN2 and CLCA1 were visualised using DAB (brown straining) in the lung epithelium. Sections were counterstained with Harris' hematoxylin (blue staining). Negative controls were carried out on serial sections by substituting the primary antibody with an isotype control (inset). Scale bar = 100 µm.

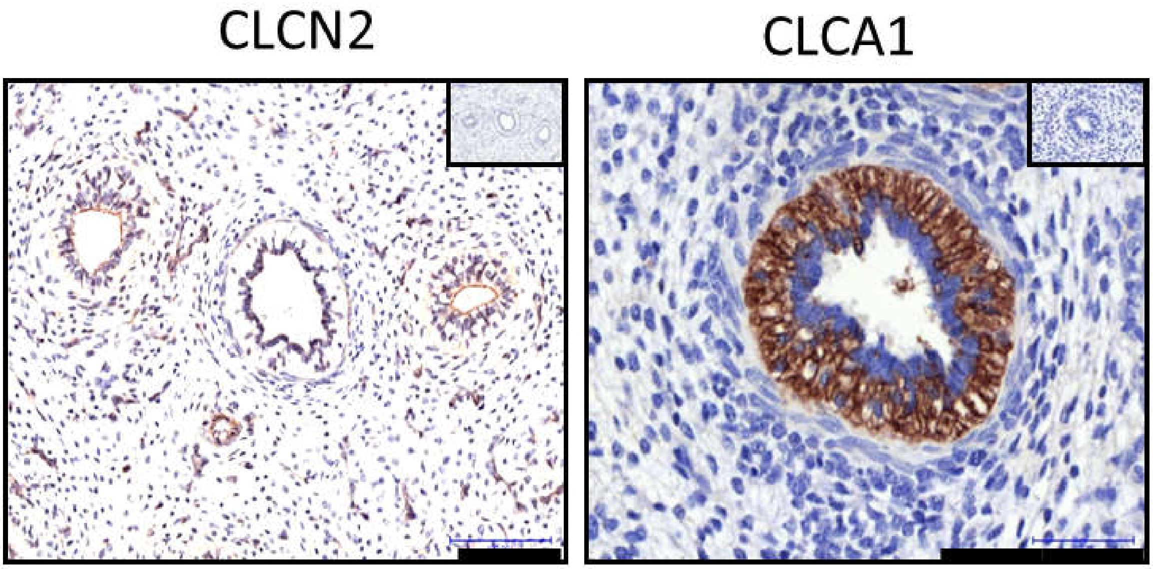

Supplement: Supplementary Information [file srep21975-s1.pdf]
